# A Label-Free and Affordable Solution to Point-of-Care Testing Devices

**DOI:** 10.3390/bios12040192

**Published:** 2022-03-24

**Authors:** Mon-Juan Lee

**Affiliations:** 1 Department of Bioscience Technology, Chang Jung Christian University, Tainan 71101, Taiwan; mjlee@mail.cjcu.edu.tw; 2 Department of Medical Science Industries, Chang Jung Christian University, Tainan 71101, Taiwan

Clinical diagnosis and disease monitoring often require the detection of small-molecule analytes and disease-related proteins in body fluids. Most conventional biochemical assays for protein detection are label-based immunoassays such as the enzyme-linked immunosorbent assay (ELISA), the gold standard of immunoassays. Fluorescence-labeled antibodies are commonly used in these bioanalytical methods to signal the formation of immunocomplexes as a result of the specific binding between an antigen and an antibody, either of which can be the target of detection. Both fluorescence labeling and fluorometric instruments increase the cost of the analysis, and limit the operation of such assays to trained medical professionals. In an era of global pandemics, the need for personal health monitoring through point-of-care testing (POCT) devices, especially during home quarantine, has become even more imperative. To develop POCT devices with lower cost that can be used more prevalently among untrained personnel, label-free detection approaches, such as electrical and electro-optical biosensing, are being investigated in the hope of enhancing detection sensitivity and increasing the linear range of detection. This Special Issue, “Electrical and Electro-Optical Biosensors”, includes six research articles covering biosensors based on electrochemical impedance, localized surface plasmon resonance, and dielectric properties of liquid crystals (LCs), as well as two review articles on printed electrochemical biosensors. The reported biosensors were designed to detect glucose, bovine serum albumin (BSA), and the cancer biomarker CA125; and to discern between different mosquito-borne viruses and cancer cells.

Surface plasmon resonance (SPR), the electron oscillation stimulated by an incident light on metal surface, is one of the most common label-free biosensing technologies. Localized SPR (LSPR) refers to SPR confined to nanoparticles with diameters comparable to the wavelength of the incident light, which is suggested to enhance detection sensitivity through signal amplification. A millimeter-wave-based spoof localized surface plasmonic resonator was developed for glucose detection in a microfluidic system [1]. The millimeter-wave-based glucose sensor exhibited higher sensitivity than microwave-based sensors with a limit of detection (LOD) of 1 mg/dL from a sample volume of 3.4 μL, and is reusable with satisfactory reproducibility. The performance of the LSPR glucose sensor is comparable to commercial glucose sensors such as the Accu-Chek blood glucose meters manufactured by Roche, which rely on electrochemical signals produced by the reaction of glucose with glucose oxidase and have a detection range of 10–600 mg/dL glucose for a sample volume ranging from 0.3 to 2 μL.

Electrical biosensors detect biological binding events occurring on the electrode and transduce electrical signals in the form of conductance, resistance, or capacitance, which are dependent on the amount of analyte. In an electrical biosensor consisting of a silicon-on-insulator nanowire immobilized with antibodies against CA125, the dependence of electric current on CA125 concentration was established [2]. The high surface-to-volume ratio of the nanowire enabled sensitive detection of CA125 to concentrations as low as 10^−16^ M. On the other hand, electrochemical impedance spectroscopy (EIS) has become a powerful tool for electrical biosensing. Biomolecular recognition resulted from antigen–antibody or ligand–receptor binding alters the electron transfer resistance at the surface of the electrode, which can then be quantitatively analyzed and correlated to the amount of analytes. An impedimetric biosensor based on a gold–polyaniline and sulfur-/nitrogen-doped graphene quantum dot nanocomposite conjugated with antibodies was designed for the detection of mosquito-borne viruses [3]. Dengue virus, zika virus, and chikungunya virus were discerned by the impedimetric biosensor with minimal cross-reactivity and LOD in the range of femtogram per milliliter. Moreover, the dielectric characteristics of several cancer cell types, represented by crossover frequencies, were determined by capturing cells on interdigitated microelectrodes through dielectrophoresis, followed by EIS analysis [4]. These results demonstrate that the selectivity or specificity of detection in electrical biosensors can be realized by including target-specific antibodies in the sensor design, or by examining the unique electrical signal produced by the analyte.

Printed circuit board (PCB) is one of the key technologies to miniaturize and lower the cost of point-of-care testing devices. It also facilitates easy integration of the sensing platform with more sophisticated electronic and microfluidic systems [5,6]. Consisting of multilayers of conductive and insulating materials, PCB was originally a component of the integrated circuit for the electronics industry [6]. In electrochemical biosensors, which transduce biochemical signals through amperometric, impedimetric, or potentiometric principles, the two- or three-electrode circuit system was printed on a small surface area by screen printing, inkjet printing, or aerosol jet printing procedures, which varies in resolution and ink dispensing methods [5]. Various printing strategies were established to enhance detection sensitivity and LOD, as well as to increase biocompatibility for the purpose of direct detection in a biological environment. With a growing demand for medical wearable devices, the increasing versatility of PCB technology was seen in the development of flexible and stretchable PCBs. Currently, printed electrochemical biosensors utilizing amperometry, cyclic voltammetry, and EIS were developed for the detection of small-molecule metabolites such as glucose and lactate, disease-related marker proteins such as interferon-gamma, DNA associated with single nucleotide polymorphism, and whole cells such as eukaryotic cells and pathogens [5,6]. With novel printing technology and fabrication procedures to improve metrological performance, including signal-to-noise ratio, detection sensitivity, repeatability, and reproducibility, PCB-based biosensors are expected to become the mainstream biosensing technologies for affordable point-of-care diagnostics.

LCs have become an indispensable material in our daily lives, seen predominantly in LC display devices such as smartphones, digital clocks, and flat-screen televisions. LCs are fluidic but exhibit molecular order similar to solid crystals, and can be induced to reorient their molecular alignment under the influence of temperature, electromagnetic radiation, electric or magnetic fields. Because the optical, electrical, and electro-optical properties of LCs are altered in a concentration-dependent manner by biological analytes, biosensing application of LCs has been extensively explored in recent decades. Conventional LC-based biodetection at the LC–glass interface was performed in a LC cell with a thin film of LCs sandwiched between a pair of glass substrates. By doping the nematic LC E7 with a prepolymer, NOA65, followed by photopolymerization to produce a LC–photopolymer composite, the optical and dielectric signal of the LC-based biosensor can be enhanced [7]. To simplify the preparation procedure of the LC–glass detection platform, a single glass substrate spin-coated with a LC film, instead of a LC cell, was utilized in the detection of BSA and CA125 [8]. Signal amplification was achieved in such single-substrate detection due to the reduced film thickness of the spin-coated LC film. Most LC-based biosensors reported to date consist of thermotropic LCs, which dominate the LC display industry. Nevertheless, lyotropic LCs, which are hydrophilic and thus more biocompatible, may hold greater potential in the biomedical application of LCs. Biosensing techniques based on the nematic phase of disodium cromoglycate (DSCG), a type of lyotropic chromonic LCs, were demonstrated in the detection of BSA and CA125 with a LOD comparable to those of nematic thermotropic LC-based biosensors [9].

Electrical and electro-optical biosensing technologies are critical to the development of innovative POCT devices, which can be used by both professional and untrained personnel to provide necessary health information within a short time for medical decision to be made, and are especially important in an era of global pandemics. This Special Issue includes some of the pioneering work on biosensors utilizing electrochemical impedance, localized surface plasmon resonance, and bioelectricity of sensing materials as the signal response that is pertinent to the amount of analyte. The results presented demonstrate the potential of these label-free biosensing approaches in the detection of disease-related small-molecule metabolites, proteins, and whole-cell entities.
